# Biofilm-derived oxylipin 10-HOME mediated immune response in women with breast implants

**DOI:** 10.1172/JCI176547

**Published:** 2024-02-01

**Authors:** Tyler M. Bauer, Katherine A. Gallagher

**Affiliations:** 1 Department of Surgery and; 2 Department of Microbiology and Immunology, University of Michigan, Ann Arbor, Michigan, USA.

## Abstract

Breast implant illness (BII) is a poorly understood disease in which patients develop symptoms typical of autoimmune conditions following breast implantation. There is no known underlying cause, and patients often resort to breast implant removal and capsulectomy to alleviate symptoms. In this issue of the *JCI*, Khan and colleagues examined 86 breast explants from patients that reported BII symptoms and 55 control explants. The BII group showed a disproportionally high degree of biofilm, which was associated with oxylipin (10-HOME) on the implant surfaces. Injections of 10-HOME in the mammary fat pad of a murine model recapitulated BII symptoms and increased Th1 cell populations. Notably, macrophages in the periprosthetic tissue from BII patients were more likely to exhibit a proinflammatory phenotype, and naive T cells exposed to 10-HOME caused naive macrophages to differentiate to a proinflammatory phenotype. This work provides a pathophysiologic mechanism for a currently understudied and poorly characterized disease.

## Breast implant illness is rare and poorly understood

While silicone was long thought to be biologically inert, there has been a growing interest in potential associations between various diseases and silicone implants. For example, breast implant–associated anaplastic large cell lymphoma (BIA-ALCL) was formally recognized by the WHO in 2016 and has brought silicone implants under closer scrutiny. Further investigation of BIA-ALCL showed that the malignancy has high association with textured implants (as opposed to smooth implants) and is, in fact, driven by chronic infection, likely secondary to biofilm production (1). Capsular contracture is also thought to be secondary to a dysregulated host response, possibly in response to chronic infection, resulting in poor cosmetic outcomes after breast implants (2). Following the identification of malignancy associated with silicone implants, interest in understanding the mechanisms by which silicone breast implants induce disease and the association of chronic infection was renewed.

While formal definitions leave much to be desired, approximately 50 possible systemic subjective symptoms are attributable to BII, including fatigue, anxiety, and chronic pain (3). In certain cases, objective signs of the disease can manifest, including endocrine, peripheral nervous system, and somatic dysfunctions (4, 5). Unlike BIA-ALCL, there is no association between breast implant type and BII (6). Currently, surgical removal of the breast implant as well as capsulectomy is the only effective treatment, and one study showed that BII accounts for approximately 4% of all implant removals (7). Laboratory testing that is sensitive or specific for BII remains to be identified (8). There are multiple competing theories as to the cause of BII, although to date none have been convincingly proven (9). Previous clinical studies have shown chronic infections are present in a high proportion of explants for BII, although these studies have been limited by the relatively low sensitivity of typical clinical microbiological testing in identifying organisms that are implicated in chronic infections (6). This data, in addition to the association between other breast implant–related diseases, such as BIA-ALCL and capsular contracture, has led to investigation into the relationships between chronic infections, biofilms, and BII (2).

## 10-HOME is noted in BII patient’s breast implants

In this issue of the *JCI*, Khan and authors present the largest translational study of human BII patients to date, observing nearly 140 total patients undergoing breast implant removal, with over half reporting BII symptoms (10). Through electron microscopy and the use of next generation sequencing, the authors show that there is a higher likelihood of *S*. *epidermidis,* among other bacteria, present on the implants of patients with BII. This data is an important addition to the literature, as previous investigations have only employed clinical microbiological testing, which has struggled to completely define the rate and types of chronic infections in BII (6). Furthermore, the authors show that (E)-10-hydroxyoctadecenoic acid (10-HOME), an oxylipin produced by bacteria that oxidize host oleic acids, is higher in the periprosthetic breast tissue of patients with BII (10). It is known that oxylipins such as 10-HOME promote the establishment of bacterial biofilms (11). The authors show that the bacteria in higher abundance in the BII group produced more 10-HOME (10).

**Figure 1:**
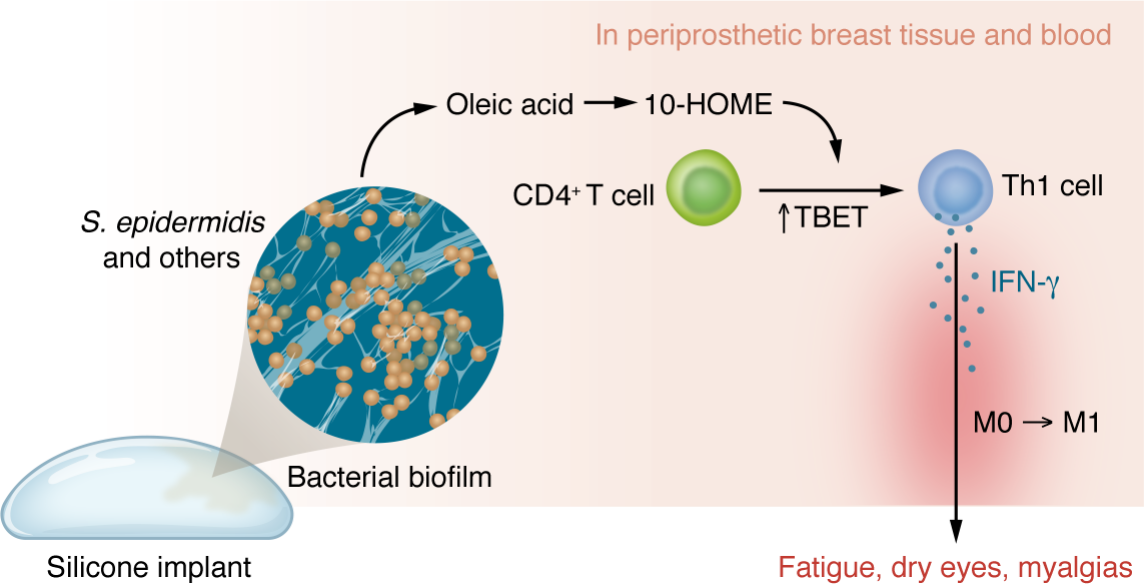
10-HOME from biofilm on implants provides a model for the immune response in women with BII. Bacteria such as *S*. *epidermidis* can establish bacterial biofilms on the surface of breast implants. *S*. *epidermidis* and/or other bacterial strains oxidize oleic acid to produce 10-HOME, which perfuses into periprosthetic breast tissue and blood. CD4^+^ T cells exposed to 10-HOME show increased expression of TBET and polarize into Th1 cells. Secretion of inflammatory factors by Th1 cells drives inflammatory macrophage polarization to yield an M1-like proinflammatory phenotype and related symptoms.

Khan and authors found through analysis of bulk RNA-Seq of the breast-implant associated tissues, that gene expression related to adaptive T cell responses, specifically the upregulation of the transcription factor TBET, was altered. This finding is important, since TBET is a transcription factor that is associated with the Th1 cell subtype. The authors followed up with a more in-depth investigation of the periprosthetic tissue, which identified a higher proportion of Th1 cells in the BII patients, and an associated increase of the Th1 cytokine IFN-γ with normal levels of Th2-associated cytokines. In vitro analysis of undifferentiated human CD4^+^ T cells showed a polarization towards the Th1 subtype with an absence of differentiation toward other T cell subtypes, and supernatants from *S*. *epidermidis* partially recapitulated the Th1 cell polarization. The authors attributed the polarization to heterogenous factors in the supernatant. Notably, mice injected with 10-HOME in the mammary fat pad showed increased CD4^+^ Th1 cells and exhibited fatigue symptoms. Finally, macrophages polarized toward an M1-like phenotype in human periprosthetic tissue, which was again noted in the murine model injected with 10-HOME. Hence, the authors suggest that Th1 cells, as a result of 10-HOME exposure, drive inflammatory macrophage polarization (10) (Figure 1).

Khan and colleagues present convincing evidence regarding the connection between the oxylipin 10-HOME and BII, which prompts us to speculate about experiments for future investigations. The most important of which will be those that define the mechanism by which macrophages polarize to a proinflammatory T cell phenotype, and its contribution to BII symptomatology. At present, the authors noted that macrophages were skewed to an M1-like proinflammatory phenotype in periprosthetic tissue, and confirmed, through coculture studies that pretreated T-cells with 10-HOME, that the polarization is likely due to T-cell related interactions (10). It is unclear at the present to what degree the inflammatory macrophage phenotype drives the autoimmune-like symptoms of BII. Future directions should seek to understand the relevancy of inflammatory macrophages to BII symptomatology. Additional studies aimed at determining the contribution of the macrophage phenotype to BII symptoms and related mechanisms may identify targets for pharmacologic treatments of BII, either through macrophage-specific therapies or T-cell directed therapies. Future efforts in silicone implant device development should be aimed at reducing the susceptibility of these devices to biofilm production in order to decrease the inflammatory immune response.

## Implications and conclusions

Khan and colleagues presented a comprehensive analysis of nearly 140 patients undergoing breast implant removal and capsulectomy and identified a potential mechanism for the development of BII (10). It appears that BII is, at the very least, associated with bacterial biofilms, and this disease is driven by chronic host response to bacterial colonization. Previous publications have failed to completely characterize biofilms in patients reporting symptoms consistent with BII (6), and the present publication emphasizes the need for advanced microbiology and pathology techniques coupled with next generation sequencing tools, inclusive of special sequencing, in order to study this rare and poorly understood disease (10). Future work is necessary to decipher the relationships between chronic infections and host response in patients that develop BIA-ALCL or BII and those that do not.

## Version 1 02/01/2024

Electronic publication

## References

[1] Lajevardi SS (2022). What are the likely causes of breast implant associated anaplastic large cell lymphoma (BIA-ALCL)?. JPRAS Open.

[2] Ajdic D (2016). The relationship of bacterial biofilms and capsular contracture in breast implants. Aesthet Surg J.

[3] Bird GR, Niessen FB (2022). The effect of explantation on systemic disease symptoms and quality of life in patients with breast implant illness: a prospective cohort study. Sci Rep.

[4] Serena TJ (2023). Breast implant illness: a cohort study. Cureus.

[5] Suh LJ (2022). Breast implant-associated immunological disorders. J Immunol Res.

[6] Lee M (2020). Breast implant illness: a biofilm hypothesis. Plast Reconstr Surg Glob Open.

[7] Lieffering AS (2022). Prevalence of local postoperative complications and breast implant illness in women with breast implants. JAMA Netw Open.

[8] Alijotas-Reig J (2018). Autoimmune/inflammatory syndrome induced by adjuvants-ASIA-related to biomaterials: analysis of 45 cases and comprehensive review of the literature. Immunol Res.

[9] de Faria Castro Fleury E, Brawer AE (2022). Fundamentals of breast implant illness and device imaging. Int J Inflam.

[10] Khan I (2024). Biofilm derived oxylipin 10-HOME–mediated immune response in women with breast implants. J Clin Invest.

[11] Martínez E, Campos-Gómez J (2016). Oxylipins produced by Pseudomonas aeruginosa promote biofilm formation and virulence. Nat Commun.

